# Correlates of Adherence of Multimodal Non-pharmacological Interventions in Older Adults With Mild Cognitive Impairment: A Cross-Sectional Study

**DOI:** 10.3389/fpsyt.2022.833767

**Published:** 2022-06-03

**Authors:** Xue Sun, Lina Wang, Xinhua Shen, Cheng Huang, Zhuqin Wei, Liming Su, Simeng Wang, Xiaoshen Liu, Xueting Zhen

**Affiliations:** ^1^School of Medicine, Huzhou University, Huzhou, China; ^2^Department of Neurosis and Psychosomatic Diseases, Third People's Hospital of Huzhou, Huzhou, China; ^3^Department of Orthopedics, Sir Run Run Shaw Hospital, Zhejiang University School of Medicine, Hangzhou, China; ^4^School of Medicine, Tongxiang City Health School, Jiaxing, China

**Keywords:** mild cognitive impairment, adherence, multimodal interventions, non-pharmacological interventions, cognitive function

## Abstract

**Background:**

Non-pharmacological interventions are promising for delaying cognitive decline in older adults with mild cognitive impairment (MCI). Although some studies have demonstrated adherence rates and factors influencing participation in single modality non-pharmacological interventions, little is known about the level and correlates of adherence to multimodal non-pharmacological interventions (MNPIs) in older adults with MCI.

**Objective:**

This study aimed to explore the adherence level and the correlates of adherence to MNPIs in older adults with MCI.

**Methods:**

A cross-sectional design was employed. Community-dwelling older adults aged 60 years and over were recruited from senior community centers and healthcare centers in Huzhou from March 2019 to December 2020. Data were collected by a general information questionnaire and the adherence scale of cognitive dysfunction management (AS-CDM) in older adults with MCI. Hierarchical regression analyses were applied to explore the correlates of adherence to MNPIs.

**Results:**

A total of 216 completed questionnaires were finally analyzed. Of these, 68.52% were female, and 45.4% of the participants had no less than 6 years of education. The overall mean score for adherence was 117.58 (SD = 10.51) out of 160, equivalent to 73.49 in the hundred-mark system, indicating a medium-level adherence to MNPIs in older adults with MCI. Of the five dimensions of adherence (AS-CDM), self-efficacy scored the highest, and the lowest was perceived barriers. The univariate analysis showed that the factors associated with the adherence to MNPIs were: regular physical exercise, meat-vegetable balance, absence of multimorbidity, high level of education, living alone, and living in urban (*p* < 0.05). In the hierarchical regression analysis, the final model explained 18.8% of variance in overall adherence (*p* < 0.01), which high school (Beta = 0.161, *p* < 0.05), college and above more (Beta = 0.171, *p* < 0.05), meat-vegetarian balance (Beta = 0.228, *p* < 0.05), regular physical exercise (Beta = 0.234, *p* < 0.05), and presence of multimorbidity (Beta = −0.128, *p* < 0.05) significantly contributed to adherence. In addition, nearly 80% of older adults with MCI preferred MNPIs.

**Conclusion:**

Early assessment and management of adherence to MNPIs were essential in older adults with MCI. Furthermore, the findings shed light on several critical areas of intervention to improve adherence to MNPIs in older adults with MCI.

**Clinical Trial Registration:**

http://www.chictr.org.cn/showproj.aspx?proj=35363, ChiCTR1900020950 (Registered on January 23, 2019).

## Introduction

Mild cognitive impairment (MCI) is an intermediate stage between normal aging and early dementia, and is mainly manifested by a progressive decline in memory or other cognitive functions (1). The overall prevalence of MCI ranges from 6.7 to 25.2% with age (2). Meanwhile, the annual progression rate from MCI to dementia varies between 8 and 15%, and 50% of people with MCI will progress to dementia within 5 years, meaning that it is an essential condition to identify and treat (3, 4). However, updated guidelines from the American Academy of Neurology (AAN) stated that no high-quality evidence exists to support pharmacological treatments for older adults with MCI (2). Given the effectiveness of non-pharmacological interventions in improving cognitive function, such as physical exercise, cognitive training, and dietary treatments, most studies are more inclined to non-pharmacological interventions (2, 5).

It cannot be denied that long-term adherence plays a crucial role in completing a variety of non-pharmacological interventions. However, older adults with MCI are physically inactive and their adherence to physical exercise is poor (6, 7). In a 1-year exercise management program (7), 41.8% of individuals completed <1/3 of this program, and 10.4% of individuals performed none of the exercise sessions, with the mean adherence of 33.2 ± 25.5%. Low motivation, lack of interest, poor health status, and lower socioeconomic status have been identified as factors influencing the adherence to exercise participation in older adults (7, 8). Although the evidence suggests that the Mediterranean Diet (MD) has a better effect on slowing cognitive decline and decreasing the risk of developing Alzheimer's disease (9), more than half of the participants (52.1%) showed low adherence to the MD in older adults (10). In contrast, higher MD adherence was significantly associated with younger age, female, higher educational level, and better anthropometric parameters (10). In addition, cognitive training provides the best non-pharmacological approach to improving cognitive function in older adults with MCI (11). Training method, duration, fatigue or other health-related limitations, and the difficulty of the cognitive tasks may affect individual adherence to cognitive training (12, 13). Therefore, there is much room for improvement in adherence to cognitive dysfunction management activities for older adults with MCI.

The adherence assessment tools are commonly applied to evaluate individuals' level of adherence at baseline or during the intervention, and indirectly to reflect the feasibility and acceptability of cognitive dysfunction management activities (14). Most previous studies relied on a single indicator/goal attainment to measure adherence to a single non-pharmacological intervention in older adults with MCI (15–18). For exercise intervention, adherence is often evaluated by the number of completions, duration of exercise, and tolerance to exercise intensity (15). A goal/task attainment scale is commonly used in cognitive training to assess adherence (16). As for diet interventions, adherence is always evaluated indirectly by the amount or frequency of food intake (17, 18). In recent years, multimodal non-pharmacological interventions (MNPIs) have become an emerging (19). A current systematic review showed that MNPIs provided more significant improvements in cognitive-motor abilities and cognitive function in older adults with MCI (20). However, the preference to MNPIs in older adults with MCI is poorly understood. Moreover, little is known about the level and correlates of adherence to MNPIs in this population. Identifying correlates of adherence to MNPIs is crucial to developing therapeutic interventions aiming to increase control, prevent adverse effects of treatment, and improve long-term outcomes.

The Health Belief Model (HBM) has been used to explain and predict to adherence with health and medical care recommendations for more than 20 years (21, 22). This model defines the key factors that explain health behaviors, including an individual's perceived threat to disease (perceived susceptibility), the belief of consequence (perceived severity), potential positive benefits of action (perceived benefits), perceived barriers to action, exposure to factors that prompt action (cues to action), and confidence in the ability to succeed (self-efficacy) (21). Researchers have used the HBM as a generalized conceptual framework to explain and predict health behaviors engagement or adherence across a spectrum of medical conditions in various subjects (23–25). In our previous work (26), we have developed the adherence scale of cognitive dysfunction management (AS-CDM) based on HBM for older adults with MCI. AS-CDM involves three kinds of non-pharmacological interventions: physical exercise, cognitive training, and dietary treatment. The AS-CDM can be used to assess the overall level of adherence to MNPIs in which individuals are currently participating or may participate in the future, in terms of both adherence behavior and adherence intentions. Meanwhile, it also used to evaluate the preferred intervention modality of cognitive dysfunction management (single/mixed modality), with good validity and internal consistency reliability (see Supplementary Materials 1). Considering that HBM allows for the inclusion of individual-level variables influences on subsequent action (27), diverse potential individual-level factors, including socio-demographic, biological (medical) and lifestyle and behavior characteristics, may influence the level of adherence to MNPIs in older adults with MCI.

Therefore, our study seeks to answer three primary questions:

(1) Whether older adults with MCI are inclined to adopt the MNPIs strategy.
(2) What is the level of adherence to MNPIs in older adults with MCI?
(3) What are the correlates that may explain the differences in adherence to MNPIs at individual-level in older adults with MCI?

## Materials and Methods

### Ethics Statement

This study was approved by the Medical Ethics Committee of the Third People's Hospital of Huzhou of Zhejiang Province, following the guidelines of the Declaration of Helsinki (reference number 2018-031) and registered at Chictr.org.cn (ChiCTR1900020950). Written informed consents were obtained from each participant. This study complied with the Strengthening the Reporting of Observational Studies in Epidemiology (STROBE) reporting guideline for cross-sectional studies (28).

### Study Design and Population

This research was a cross-sectional study conducted in Huzhou city, Zhejiang, China. The participants were included: (1) age ≥ 60 years old; (2) diagnosed of MCI; (3) absence of self-reported visual or auditory impairment; and (4) able to make an informed consent. A trained neurologist-psychiatrist made the evaluation based on the diagnostic criterion for MCI (29). The following were adopted as MCI operational criteria at screening: (1) report of a relative decline in cognitive functioning during the past year by the participant or informant; (2) normal general cognitive function, including a Beijing version of Montreal Cognitive Assessment score (15< MoCA score was < 26) (30, 31); (3) intact activities of daily living (ADL score was < 16) (32); (4) absence of dementia, including a Mini-Mental State Examination (MMSE) score of 25-30 (33).

The exclusion criteria of the study were: (1) a history of neurological, psychiatric, and other severe medical issues that may affect brain function; (2) a history of alcohol dependency or other addiction within 10 years; (3) taking any medications in the past 6 months which may cause impaired or improved cognitive performance.

### Sample Size Determination

The sample size was calculated by considering the assumptions for the single population proportion. The prevalence of MCI in China of the previous study was 14.7% (34), the overall rate is 14.7% (π = 0.147). The allowable error is 5% (δ = 0.05), and u_α/2_ is the 1 –_α/2_ percentile of the standard normal distribution, which is 1.96 when α = 0.05 (α = 0.05, u_α/2_ = 1.96). Therefore, the following formula: n = ($\mu_{\text{α}/2}^{2}\pi$[1 – π])/δ^2^= (1.96^2*^0.147^*^0.853)/0.05^2^=192. Meanwhile, considering invalid questionnaires and expanding the sample size by 10%, the total sample size was 211.

### Recruitment Process

We recruited a convenience sample of community-dwelling older adults aged 60 years and over from senior community centers and healthcare centers in Huzhou from March 2019 to December 2020. Our staff distributed recruitment leaflets at community health centers and local senior centers. Local healthcare providers also helped to make referrals to our study. Word of mouth resulted in the recruitment of older adults as well. In addition, our study team conducted free health lectures and screening events on cognitive impairments to attract interested populations. Individuals who showed interest were invited for an in-person interview to screen for eligibility by three trained staff. In the first round of eligibility assessments, a total of 832 potential participants were recruited with complaints of memory impairment. The second round of eligibility assessments included basic demographic data, functional assessment of daily activities, general cognitive function, medical history. A trained neurologist-psychiatrist made the evaluation based on the diagnostic criterion for MCI. Finally, 583 individuals were excluded due to ineligibility, and 249 eligible participants were included in the study.

### Variables and Measurements

#### Variables and Measurements for Analyzing

##### General Information

The general information questionnaire was designed by researchers, which had a tripartite structure: (1) socio-demographic information (e.g., sex, age, marital status, education, living conditions, income); (2) lifestyle information, including eating habits, physical exercise, smoking status, alcohol consumption; and (3) medical characteristics, such as family history of Alzheimer's and presence of multimorbidity. Details of the names and value assignments of each variable were described in Supplementary Materials 2.

##### Adherence of MNPIs

The adherence scale of cognitive dysfunction management (AS-CDM) was used to assess the overall level of adherence to MNPIs in which individuals are currently participating or may participate in the future, in terms of both adherence behavior and adherence intentions (26). The AS-CDM composes six dimensions: perceived susceptibility to MCI (7 items), perceived severity to MCI (4 items), perceived benefits of cognitive dysfunction management of MNPIs (3 items), perceived barriers to performing the cognitive dysfunction management (7 items), cues to complete the cognitive dysfunction management (3 items), self-efficacy (8 items), 32 items in total with higher scores indicating better adherence of cognitive dysfunction management. The content validity of AS-CDM was 0.92 according to the expert consultation and empirical analysis, and the Cronbach's α coefficient was 0.904.

#### Variables and Measurements for MCI Screening

##### Cognitive Function and Physical Function Status

The Beijing Version of the Montreal Cognitive Assessment Test (MoCA) was administered to assess global cognitive function (30). The MoCA tests includes analysis of visuospatial, executive ability, naming, attention, memory, language, abstraction, and orientation skills, aggregated for a maximum score of 30. Its scores range from 0 to 30, with a higher number indicating better cognitive performance. By using a cut-off score of 26, it gives the optimal sensitivity (92.4%) and specificity (88.4%) in distinguishing between older adults with MCI and those with intact cognitive function (35).

The Chinese version of the Lawton and Brody's Activities of Daily Living Scale was used to assess physical functional status (32). The scale consists of the Physical Self-Maintenance Scale (PSMS) with six questions and the Instrumental Activities of Daily Living Scale (IADL) with eight questions. The scores range from 14 to 56, with a higher score indicating a lower level of ADL functioning. A score of <16 means that older adults with MCI have their activities of daily living intact. The Cronbach's α coefficient was 0.93 and Split-half coefficient was 0.91; the range of intraclass correlation coefficient of the 14 items with total score of the scale was 0.84–0.96 (36).

### Data Collection

Data were collected using a structured questionnaire through a face-to-face interview. Trained health professionals facilitated data collection and supervision to ascertain the quality of data. The training was given to data collectors and supervisors for 2 days duration to minimize measurement bias. Furthermore, the older adults with MCI completed the questionnaires about 30 min. We gave a small gift to those who completed this survey as compensation.

### Statistical Analysis

Data were analyzed in SPSS 25.0 (SPSS Inc., Chicago, IL, USA). Normally distributed continuous variables were expressed as mean ± standard deviation (SD), and categorical variables were presented as frequency and percentage. Differences in the level of adherence between/among groups by each variable were analyzed by independent samples *t*-test or ANOVA. Hierarchical regression analyses were further conducted to identify the correlates of adherence to MNPIs for older adults with MCI. In the first model (model A), socio-demographic variables were included; In the second model (model B), the lifestyle information was entered; The disease-related factors were entered in the last model (model C). The level of statistical significance was set at *p* < 0.05.

## Results

### Descriptive Data of Participant Characteristics

**Figure 1:**
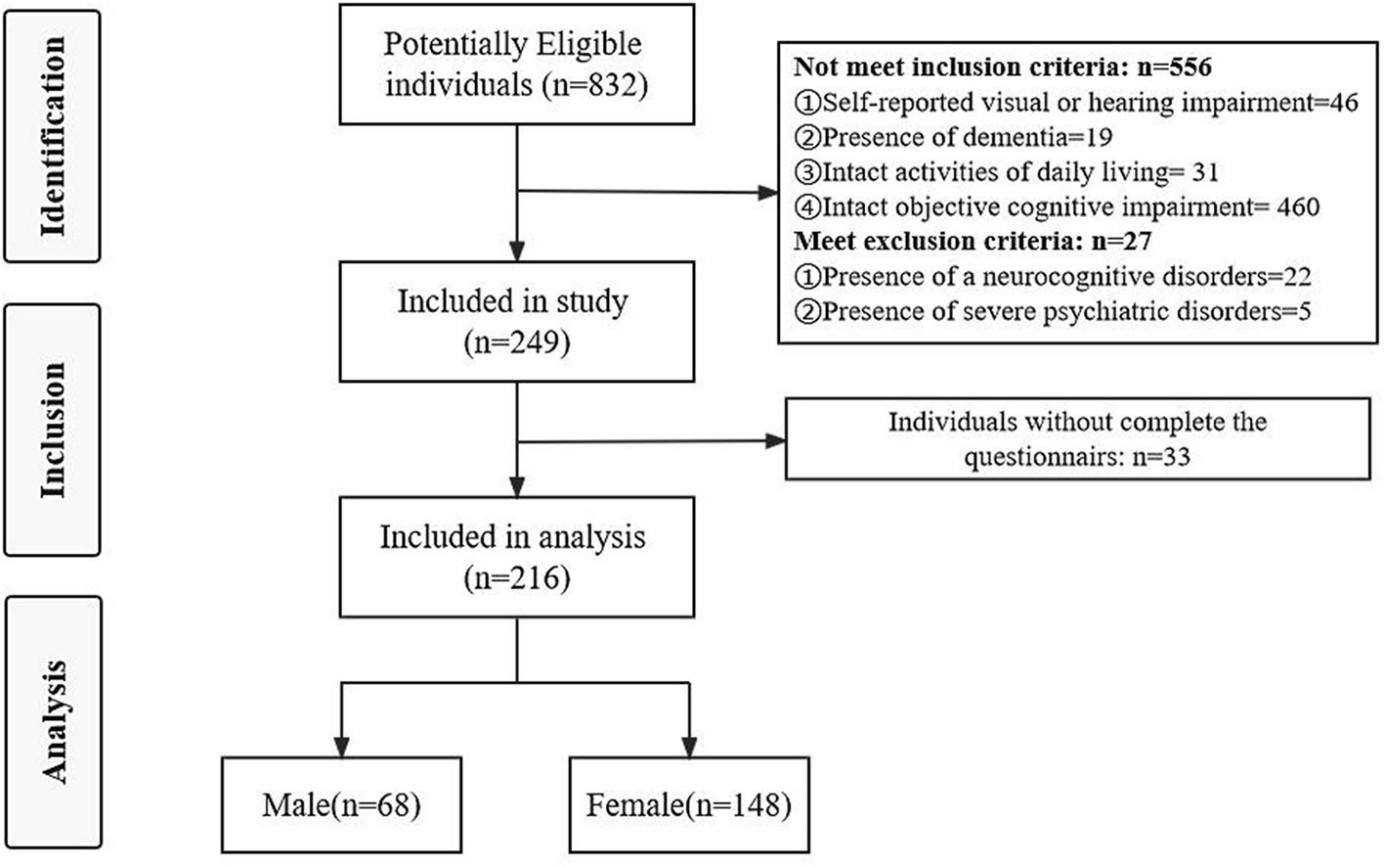
Flow diagram of participants recruitment for a cross-sectional study.

The flow diagram (Figure 1) shows 249 participants were included in this study and 33 did not complete the questionnaire. Finally, a total of 216 completed questionnaires were finally analyzed. Of these, 68.52% were female, and 45.4% of the participants had no <6 years of education, and only 6% had more than 12 years of education. Less than 15% of the participants were single (divorced/widowed), and 14.4% lived alone. More than 75% of the participants were engaged in physical labor, and most of the participants have a monthly income of <3,000. The detailed demographic data are summarized in Table 1.

**Table 1:** Characteristics of the study participants (*N* = 216).

**Characteristics**	***n***	**(%)**	**Characteristics**	***n***	**(%)**
**Sex**			**Region of residence**		
Male	68	31.48	Urban	136	63.00
Female	148	68.52	Rural	80	37.00
**Race**			**Living conditions**		
Han	194	89.80	Living alone	31	14.40
Minority	22	10.20	Living with others	185	85.60
**Marital status**			**Current smoker**		
Married	185	85.60	Yes	46	21.30
Single (divorced, widowed)	31	14.40	No	170	78.70
**Age**			**Alcohol consumption**		
60–69	147	68.06	Yes	55	25.50
70–79	58	26.85	No	161	74.50
≥80	11	5.09	**Eating habits**		
**Education level**			Meat-vegetable balance	144	66.70
Primary school and below	98	45.40	Meat-based	16	7.40
Junior school	60	27.80	Vegetarian based	56	25.90
High school	45	20.80	**Physical exercise**		
College and above	13	6.00	Never	26	12.00
**Nature of occupation**			Regular^^**#**^^	152	70.40
Mental labor	54	25.00	Irregular	38	17.60
Physical labor	162	75.00	**Family history of AD**		
**Monthly income**			Yes	17	7.90
<1,000	43	19.90	No	199	92.10
1,001–2,999	100	46.30	**Presence of multimorbidity**		
3,000–5,999	62	28.70	Yes	134	62.00
≥6,000	11	5.10	No	82	38.00
**MoCA** (mean, SD)	19.82 ± 3.28		**ADL** (mean, SD)	14.06 ± 0.37	

*AD, Alzheimer's Disease;*

# *regular exercise means exercise with a frequency of at least twice a week and at least 30 min per session (30)*.

### Adherence Characteristics to MNPIs Among Participants

The overall mean score of AS-CDM was 117.58 (SD = 10.51) out of 160. Females had higher AS-CDM compared to males. As shown in Table 2, statistically significant differences in adherence were distributed by education level (*p* = 0.001), living conditions (*p* = 0.048), registered residence (*p* = 0.029), presence of multimorbidity (*p* = 0.034), eating habits (*p* = 0.003), and physical exercise (*p* = 0.003). Participants with the following characteristics had better adherence, including regular physical exercise, meat-vegetable balance, absence of multimorbidity, high education level, living alone, and living in urban.

**Table 2:** Univariate analysis of adherence of cognitive dysfunction management (*N* = 216).

**Characteristics**	**Adherence scores**	***t/F***	***P*** **value**	**Characteristics**	**Adherence scores**	***t/F***	***P*** **value**
**Sex**				**Living conditions**			
Male	116.79 ± 11.03	−0.74	0.460	Living alone	121.03 ± 10.17	1.99	0.048^*^
Female	117.94 ± 10.28			Living with others	117.00 ± 10.48		
**Race**				**Registered of residence**			
Han	117.77 ± 10.88	0.79	0.433	Urban	118.77 ± 10.24	2.20	0.029^*^
Minority	115.91 ± 6.37			Rural	115.55 ± 10.71		
**Age group**				**Current smoker**			
60–69	117.68 ± 10.78	0.12	0.890	Yes	115.67 ± 14.16	−1.10	0.277
70–79	117.60 ± 9.28			No	118.09 ± 9.27		
≥80	116.09 ± 13.60			**Alcohol consumption**			
**Education level**				Yes	117.51 ± 10.52	−0.15	0.880
Primary school and below	114.67 ± 10.25	6.08	0.001^*^	No	117.76 ± 10.59		
Junior school	118.78 ± 10.22			**Eating habits**			
High school	120.27 ± 9.92			Meat-vegetable balance	119.23 ± 10.53	5.90	0.003^*^
College and above	124.62 ± 10.00			Meat-based	116.13 ± 7.86		
**Marital status**				Vegetarian based	113.75 ± 10.21		
Married	117.70 ± 9.58	0.40	0.686	**Physical exercise**			
Single (divorced/widowed)	116.87 ± 15.11			Never	111.35 ± 11.42	6.04	0.003^*^
**Nature of occupation**				Regular	118.84 ± 10.06		
Mental labor	119.30 ± 10.72	1.39	0.166	Irregular	116.79 ± 10.34		
Physical labor	117.01 ± 10.41			**Presence of multimorbidity**			
**Monthly income**				Yes	116.40 ± 10.90	−2.13	0.034^*^
<1,000	115.93 ± 9.64	1.60	0.191	No	119.51 ± 9.61		
1,001–2,999	116.90 ± 10.55			**Family history of AD**		
3,000–5,999	119.97 ± 10.86			Yes	118.71 ± 10.94	0.46	0.650
≥6,000	116.72 ± 10.51			No	117.48 ± 10.50		

*AD, Alzheimer's Disease;*

* *p < 0.05; Multimorbidity: two or more chronic conditions (31). The bold underline only serves to emphasize meaningful variables*.

### Correlates of Adherence to MNPIs in the Hierarchical Regression Analysis

According to Model 1 in Table 3, socio-demographic factors accounted for 10.2% of the variance of adherence (*p* < 0.001). Of these, junior school (Beta = 0.159, *p* < 0.05), high school (Beta = 0.206, *p* < 0.05), college and above more (Beta = 0.216, *p* < 0.05), living alone (Beta = 0.133, *p* < 0.05) had significant effects on the variance of adherence in model 1. After controlling for socio-demographic variates, lifestyle factors further significantly accounted for 17.2% of the variance of adherence in model 2. Physical exercise and eating habits were associated with AS-CDM scores, which explained variance increased by 7% (R^2^ change = 0.07, *p* < 0.001). Adding disease-related factors to model 3 accounted for an additional 1.6% of the variance of adherence (*p* < 0.001). In model 3, high school (Beta = 0.161, *p* < 0.05), college and above more (Beta = 0.171, *p* < 0.05), meat-vegetarian balance (Beta = 0.228, *p* < 0.05), regular physical exercise (Beta = 0.234, *p* < 0.05), and presence of multimorbidity (Beta = −0.128, *p* < 0.05) contributed significantly to adherence. The final model explained 18.8% of the total variance of adherence to MNPIs in older adults with MCI.

**Table 3:** Hierarchical regression analysis of influencing factors of adherence (*N* = 216).

**Variable**	**Model 1**			**Model 2**			**Model 3**		
	**B (SE)**	**Beta**	**95% CI**	**B (SE)**	**Beta**	**95% CI**	**B (SE)**	**Beta**	**95% CI**
**Education level (ref. = Primary school and below)**									
Junior school	3.72 (1.75)	0.159^*^	[0.27, 7.16]	2.85 (1.71)	0.122	[−0.52, 6.22]	2.43 (1.71)	0.104	[−0.94, 5.81]
High school	5.31 (1.87)	0.206^*^	[1.63, 8.99]	4.12 (1.86)	0.160^*^	[0.46, 7.78]	4.16 (1.84)	0.161^*^	[0.52, 7.80]
College and above	9.53 (3.02)	0.216^*^	[3.58, 15.47]	7.7 (2.96)	0.175^*^	[1.86, 13.54]	7.56 (2.94)	0.171^*^	[1.76, 13.36]
**Registered of residence (ref. = Rural)**									
Urban	1.28 (1.52)	0.059	[−1.72, 4.29]	1.26 (1.52)	0.058	[−1.75, 4.27]	1.43 (1.52)	0.066	[−1.56, 4.42]
**Living conditions (ref. = Living with others)**									
Living alone	3.99 (1.97)	0.133^*^	[0.11, 7.88]	4.05 (1.92)	0.135^*^	[0.26, 7.84]	3.68 (1.92)	0.123	[−0.11, 7.46]
**Eating habits (ref. = Vegetarian based)**									
Meat-based				4.39 (2.88)	0.110	[−1.29, 10.07]	4.58 (2.86)	0.114	[−1.06, 10.23]
Meat-vegetable balance				5.06 (1.55)	0.227^*^	[2.00, 8.12]	5.0 8(1.54)	0.228^*^	[2.04, 8.12]
**Physical exercise (ref. = Never)**									
Regular				5.39 (2.16)	0.235^*^	[1.14, 9.65]	5.37 (2.14)	0.234^*^	[1.15, 9.59]
Irregular				3.14 (2.56)	0.114	[−1.90, 8.18]	3.45 (2.54)	0.125	[−1.57, 8.47]
**Presence of multimorbidity (ref. = No)**									
Yes							−2.76 (1.39)	−0.128^*^	[−5.49, −0.02]
**R** ^2^		**10.2%**			**17.2%**			**18.8%**	
**R**^2^ **change (p)**		***P*** **< 0.001**			**7% (*p* < 0.001)**			**1.6% (*p* < 0.001)**	

*B, unstandardized coefficient; SE, standard error; Beta, standardized coefficient. The bold underline only serves to emphasize meaningful variables*.

* *p < 0.05*.

### Analysis of Variables That May Be Associated With Dimensions Scores of Adherence

**Figure 2:**
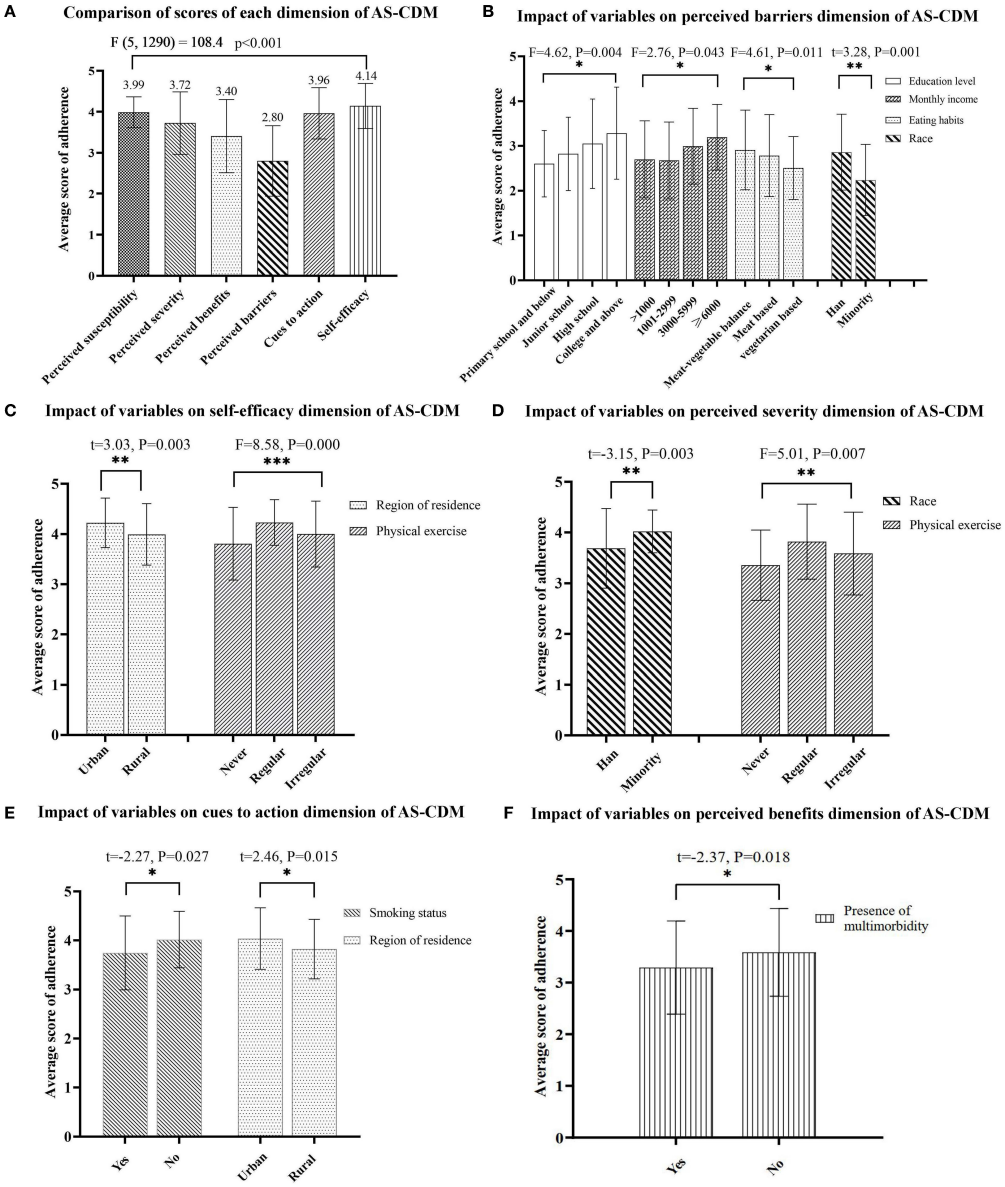
**(A–F)** Analysis of variables associated with dimensions scores of adherence. ^*^p < 0.05, ^**^p < 0.01, ^***^p < 0.001.

The average total score of the AS-CDM for all participants was 117.58 ± 10.51. Of these, the average score of perceived susceptibility dimension was 3.99 ± 0.38, perceived severity was 3.72 ± 0.76, perceived benefits was 3.40 ± 0.89, perceived barriers was 2.80 ± 0.86, cues to action was 3.96 ± 0.63, and self-efficacy was 4.14 ± 0.55 (see Figure 2A). There was a statistically significant difference in the six dimensions of the AS-CDM (*F* = 108.4, *p* < 0.001). The lowest score was for the dimension of perceived barrier. It can be significantly influenced by educational level, monthly income, eating habits, and race (see Figure 2B). In contrast, self-efficacy was the highest scoring of the adherence dimensions, and participants who had regular physical exercise and lived in the urban showing greater self-efficacy on the AS-CDM assessment (see Figure 2C). The influence factors with statistically significant of other dimensions are shown in Figures 2D–F.

### The Preferred Intervention Modality of Cognitive Dysfunction Management

The dimension of self-efficacy in the AS-CDM was used to evaluate the preferred modality of cognitive dysfunction management in this study. Of these, items 1 and 2 indicate the preference of exercise intervention, items 3 and 4 represent preference for the cognitive training intervention, and items 5–8 for preference on the dietary intervention. Each item was scored on a 5-point Likert scale; a score of ≥4 was satisfied simultaneously for all items of a specific intervention preference, representing a preference for this intervention method. On the contrary, any item score < of 4 points is defined as uncertainty. In this study, the preferred intervention modality can be classified as single modality, mixed modality, and uncertain by scoring of the specific intervention modality. As shown in Table 4, nearly 80% of older adults with MCI preferred a mixed modality of non-pharmacological interventions (i.e., MNPIs).

**Table 4:** Preferred intervention modality of cognitive dysfunction management (*N* = 216).

**Preferred mode of intervention**	**Number**	**Preference rate**
Single modality	32	14.81%
Mixed modality	172	79.63%
Uncertain	12	5.56%

*Single Modality, prone to one of the interventions among physical exercises, cognitive training, or dietary treatment; Mixed Modality, incline to two or more interventions of cognitive dysfunction management; Uncertain, no preference for any specific intervention*.

## Discussion

### Summary of the Findings

Precise identification of contributors to low adherence will be crucial for improving treatment effectiveness and distinguishing individuals needing additional supervision to decrease the risk of disease or complications (37). To our knowledge, this is the first study that examined the level and correlates of adherence to MNPIs in older adults with MCI. The adherence to MNPIs was medium-level for older adults with MCI in this study. Our findings showed significant differences in the adherence to MNPIs in the education level, physical exercise, eating habits, and multimorbidity status of participants.

### Comparison With Other Studies

#### The Adherence to Non-pharmacological Interventions for Older Adults With MCI

Whether single modality or multimodal interventions, treatment adherence might be different across the project categories. In previous RCT studies, most participants could adhere to a specific treatment during the supervised intervention; however, the level of adherence declined over the long term (7, 14). In a study of memory support systems training for older adults with MCI (14), participants in the intervention group had the highest adherence at the end of the training; however, it was consistently declining at the 8-week and 6-month follow-up visit after training. Our results showed a medium-level of adherence to MNPIs, a similar level of adherence reported by Mosca (65.5%) (38) and Lam (79%) (39). Notably, the cross-sectional data analysis in this study may overestimate the adherence of participants. Nevertheless, the present study extends our understanding of the evaluation of adherence to MNPIs in older adults with MCI.

#### Correlates of Adherence to MNPIs

The hierarchical regression analysis in this study mainly confirmed that higher level of education was associated with higher adherence to MNPIs in older adults with MCI. Previous research have also shown that higher level of education significantly predicted higher post-intervention adherence to behavioral intervention in older adults with MCI (40). Individuals with higher education may be more effective in understanding the potential benefits and actively overcoming barriers to adherence to health interventions (40). This reasonable explanation is also confirmed by the results shown in Figure 2B of this study. What's more, higher education attainment may be associated with higher cognitive reserve, which may prevent further cognitive decline and ultimately delay the onset of dementia (41, 42). Therefore, individuals with higher education may have less cognitive impairment and be more able to adhere to interventions (40). Conversely, this finding suggests that those with lower levels of education may require substantially more programmatic support to be successful with interventions (40). In this study, only 6% of participants received more than 12 years of education, which is consistent with the distribution characteristics of the education level of older adults with MCI in China (43), indicating that most older adults with MCI have a lower level of education and poorer adherence to MNPIs. Therefore, community healthcare providers should give more attention to the less educated older adults with MCI. Regular reminders and encouragement are necessary to enforce good adherence and further optimize the benefits of MNPIs.

Physical exercise and meat-vegetable balance were identified to have a significant positive association with adherence in older adults with MCI in model 2. Consistent with previous studies (44, 45), these data suggest that the level of adherence to the MNPIs was higher among participants with regular physical exercise than among those with non-regular physical exercise. It is noteworthy that the scores of both self-efficacy and perceived severity dimensions were significantly different in varying exercise states (shown in Figures 2C,D). By inference, regular exercise may improve the individuals' adherence to MNPIs by boosting their self-efficacy. Many previous studies have demonstrated the cognition-enhancing effects of exercise in older adults with MCI (46, 47). Therefore, it is recommended that primary healthcare providers should facilitate physical exercise recommendations and customize exercise programs for individuals at high risk of developing MCI.

The study results confirm that eating habits were significantly correlated with adherence to MNPIs in older adults with MCI. Participants with a meat-vegetables balance habit showed better adherence than those who ate only meat or a predominantly vegetarian diet. This finding may be attributed to the balance of meat-vegetables as a healthy diet model, similar to the Mediterranean diet composition. The latter has been proved to be an independent protective factor for cognitive function (5). The similarity of these two dietary patterns may promote the scores of diet intervention adherence of MNPIs in older adults with MCI.

In addition, the results of this study identified the status of live-alone as an independent contributor to high adherence to MNPIs. Picorelli et al. also found that living alone was associated with better adherence to exercise programs for older adults (8). This interaction can be interpreted in one of three ways: (1) Those who live alone may not have any other family members to share household responsibilities that require energy expenditure (48). (2) Older adults living alone may have complex health needs and are more likely to be the highest users of health care services (49), including MNPIs. (3) Additionally, interventions that increase social engagement (such as physical exercise, cognitive training, and dietary treatment) may reduce loneliness (50), which led to high adherence to MNPIs among participants living alone in this study. Conversely, older adults with MCI who live with family exhibit poor adherence. Therefore, it is necessary to encourage individuals and their family members to make decisions to engage in meaningful daily activities.

The final regression model illustrated that participants with multimorbidity had lower adherence to MNPIs. Adverse effects of medications may worsen cognitive function and further impede participation in MNPIs in older adults with MCI (51). Additionally, functional limitations caused by somatopathy may also diminish the adherence to MNPIs (8). This reasonable explanation is also supported by the findings shown in Figure 2F of this study. Therefore, healthcare providers need to ensure safe and effective medication administration via health education for older adults. Meanwhile, strategies to guide or reduce the use of potentially inappropriate medications (PIMs) should be implemented to minimize their adverse effects on cognitive/physical function in older adults with MCI (52). Furthermore, physical function assessment and implementation of functionally matched MNPIs are necessary for older adults with MCI suffering from multimorbidity.

#### The Preferred Intervention Mode of Cognitive Dysfunction Management

Most of older adults with MCI were inclined to MNPIs strategy in this study. A minority of participants may select a single modality due to lack of interest, health-related restrictions, and family diet plans. Twelve of these participants had no clear preference for any specific intervention. This may be partly explained by individual differences in the level of healthcare consciousness in the management of cognitive impairment. For this reason, healthcare providers should strengthen cognitive-related health education to facilitate the self-consciousness of cognitive function management and behavior adherence in older adults with MCI.

### Study Strengths and Implications

The most important strength of this study is evaluating the level and correlates of adherence to MNPIs in older adults with MCI. This topic has significant implications in screening at-risk individuals with poor adherence to cognitive dysfunction management and ineffectively promoting their cognitive-health behavior. This study also provides evidence that a high educational level, meat-vegetarian balanced diet habits, regular physical exercise, and absence of multimorbidity are associated with high adherence to MNPIs in older adults with MCI, indicating the potential targets of adherence-promoting interventions. The new findings in this study, although preliminary, could provide more valuable information to formulate guidelines on the operationalization and implementation of MNPIs for older adults with MCI.

### Limitations and Future Research Directions

The findings in this study should be understood within the context of several limitations. (1) The study location was only in Huzhou city, China, with relatively small sample size, and the generalizability of the findings is limited. Meanwhile, the convenience sampling used in this study makes the results of this study potentially susceptible to sampling bias. A large sample size, random sampling, and multi-center study are needed in the future. (2) This study was a cross-sectional design, which is unsuitable for examining causality, and longitudinal studies on MNPIs intervention for older adults with MCI are needed to determine these findings. (3) The final model in this study demonstrates that the included variables did not account for all the variance of adherence (18.8%). According to the Multidimensional Adherence Model (53), therefore, further exploration of the contribution of variables related to the healthcare system (patient-provider relationship) and other potential variables (the duration of the intervention, fatigue, social network size, social support, depressive symptomatology) is needed to formulate a more comprehensive attribution model of adherence to MNPIs for older adults with MCI.

## Conclusion

Positive effects of MNPIs have been reported and are promising for delaying cognitive decline. This study provides the first illustration of the level and correlates of adherence to MNPIs in older adults with MCI. Older adults with MCI included in this study presented medium-level adherence, although most participants were inclined to MNPIs strategy. Education level, eating habits, physical exercise, and multimorbidity status were associated with MNPIs adherence in older adults with MCI. These findings suggest the need for education on MNPIs and the potential directions for health promotion programs for older adults with MCI. Furthermore, the above correlation will help screen high-risk MCI individuals with low adherence to NMPIs, and provide a more proactive and precise intervention program.

## Data Availability Statement

The original contributions presented in the study are included in the article/Supplementary Materials, further inquiries can be directed to the corresponding author/s.

## Author Contributions

LW: devised research concept, research design, and critical revision of the manuscript for intellectual content. XSu: drafted the first version of the manuscript, submitted the manuscript for publication, and assisted in the development of the research design. XSh: contributed to the design of the study. CH, ZW, and LS: assisted with participant enrolment and consenting and data acquisition plan. SW, XL, and XZ: assisted with statistical analytic planning. All authors contributed to the design and drafting of the manuscript and read and approved the final manuscript.

## Funding

This study was supported by the National Natural Science Foundation of China (Nos. 72174061 and 71704053), the China Scholarship Council Foundation (No. 201908330251), and the Zhejiang Provincial College Students Scientific and Technological Innovation Activities-Zhejiang Xinmiao Talents Program (No. 2021R431031).

## Conflict of Interest

The authors declare that the research was conducted in the absence of any commercial or financial relationships that could be construed as a potential conflict of interest.

## Publisher's Note

All claims expressed in this article are solely those of the authors and do not necessarily represent those of their affiliated organizations, or those of the publisher, the editors and the reviewers. Any product that may be evaluated in this article, or claim that may be made by its manufacturer, is not guaranteed or endorsed by the publisher.
