# Supplementary material for: Correlates of Adherence of Multimodal Non-pharmacological Interventions in Older Adults With Mild Cognitive Impairment: A Cross-Sectional Study
Source: Front Psychiatry. 2022 Jun 3;13:833767. doi: 10.3389/fpsyt.2022.833767 (PMC9210931; doi:10.3389/fpsyt.2022.833767)
Supplement: Supplementary file 1 [file Table_1.docx]

Supplementary Material

# Supplementary Material 1

**Adherence Scale of** **Cognitive Dysfunction Management (AS-CDM) for Older Adults with Mild Cognitive Impairment**

Guidance: The following questions evaluate the adherence to cognitive dysfunction management for older adults with MCI. Please choose the answer that best suits you and tick the corresponding blank box after each question.

| **Dimension** | **Item** | **Strongly Disagree** | **Disagree** | **Uncertain** | **Agree** | **Strongly Agree** |
| --- | --- | --- | --- | --- | --- | --- |
|  |  | **1** | **2** | **3** | **4** | **5** |
| **Perceived susceptibility**  To what extent do you agree with the following statements regarding the likelihood of MCI progressing to dementia? | 1. The older you are, the more likely to develop dementia. |  |  |  |  |  |
|  | 2. If you have a family history of dementia, you are more likely to get dementia. |  |  |  |  |  |
|  | 3. Any combination of hypertension, diabetes, and hyperlipidemia is more likely to cause dementia. |  |  |  |  |  |
|  | 4. People will be more likely to develop dementia if they have noticed a significant memory decline. |  |  |  |  |  |
|  | 5. If people have the habit of smoking, they will be more likely to develop dementia. |  |  |  |  |  |
|  | 6. If people drink excessively, they will be more likely to develop dementia. |  |  |  |  |  |
|  | 7. Lack of cognitive dysfunction management is more likely to develop dementia. |  |  |  |  |  |
|  | | | | | | |
| **Perceived severity**  Which of the following adverse effects do you think mild cognitive impairment will bring to you? | 8. A decline in cognitive function affects self-care ability in daily life (e.g., handling finance, taking a car, doing housework, and using household appliances). |  |  |  |  |  |
|  | 9. Compared with patients without psychiatric symptoms (apathy, depression, anxiety, etc.), patients with psychiatric symptoms are more likely to develop dementia. |  |  |  |  |  |
|  | 10. Continuous decline of cognitive function will affect social communication ability. |  |  |  |  |  |
|  | 11. If the disease continues to develop, it will bring enormous economic pressure to the family. |  |  |  |  |  |
|  | | | | | | |
| **Perceived benefits**  What do you think is the benefit of cognitive function management for you? | 12. Help to delay the process of cognitive decline. |  |  |  |  |  |
|  | 13. Help to maintain the current level of cognitive function. |  |  |  |  |  |
|  | 14. Help to improve partially impaired cognitive function. |  |  |  |  |  |
|  | | | | | | |
| **Perceived barriers^#^**  To what extent do you agree with the following factors that hinder the implementation of cognitive dysfunction management? | 15. No free time for cognitive dysfunction management. |  |  |  |  |  |
|  | 16. No improvement in cognitive dysfunction in the short term. |  |  |  |  |  |
|  | 17. Low mood. |  |  |  |  |  |
|  | 18. Poor physical condition. |  |  |  |  |  |
|  | 19. Long time consuming per cognitive dysfunction management. |  |  |  |  |  |
|  | 20. Lack of interest. |  |  |  |  |  |
|  | 21. The authority of cognitive dysfunction management project instructors is not high. |  |  |  |  |  |
|  | | | | | | |
| **Cues to action**  To what extent do you agree with the following factors contributing to the implementation of cognitive dysfunction management? | 22. My friends and relatives have dementia. |  |  |  |  |  |
|  | 23. My memory decreased obviously. |  |  |  |  |  |
|  | 24. If the doctor suggests that I actively participate in the management of the cognitive function. |  |  |  |  |  |
|  | | | | | | |
| **Self-efficacy**  How much confidence do you have for standardized cognitive dysfunction management projects? | 25. I can do exercise twice a week (walking, jogging, aerobics, dancing, Tai Chi, etc.). |  |  |  |  |  |
|  | 26. I can stick to 30 minutes of exercise every time (walking, jogging, aerobics, dancing, Tai Chi, etc.). |  |  |  |  |  |
|  | 27. I can persist in cognitive dysfunction training (memory, orientation, computational training, etc.) 5 ~ 6 times a week. |  |  |  |  |  |
|  | 28. I can adhere to the cognitive dysfunction training of 1 hour each time (memory, orientation, computational training, etc.). |  |  |  |  |  |
|  | 29. I can keep eating 300 ~ 500 g of vegetables a day. |  |  |  |  |  |
|  | 30. I can insist on eating beans twice a week or more. |  |  |  |  |  |
|  | 31. I can insist on a monthly intake of red meat (pork, beef, mutton, etc.) not exceeding 340 ~ 450g. |  |  |  |  |  |
|  | 32. I can adhere to the staple food of whole grains (wheat, rice, maize, etc.) five times a week or more. |  |  |  |  |  |

**^#^**This dimension item needs reverse scoring.
