# Supplementary material for: Correlates of Adherence of Multimodal Non-pharmacological Interventions in Older Adults With Mild Cognitive Impairment: A Cross-Sectional Study
Source: Front Psychiatry. 2022 Jun 3;13:833767. doi: 10.3389/fpsyt.2022.833767 (PMC9210931; doi:10.3389/fpsyt.2022.833767)
Supplement: Supplementary file 2 [file Table_2.DOCX]

Supplementary Material

# Supplementary Material 2

**Table S1.** Variable name and value assignment.

|  | **No** | **Variable name** | **Variable Definitions** |
| --- | --- | --- | --- |
| Socio-demographic information | Var1 | Gender | 1= Male, 2 = Female |
|  | Var2 | Race | 1 = Han, 2 = Minority |
|  | Var3 | Age Group | 1 = 60-69, 2 = 70-79, 3 = ≥80 |
|  | Var4 | Marital Status | 1 = Married, 2 = Single(divorced/widowed) |
|  | Var5 | Nature of Occupation | 1 = Mental labor, 2 = Physical labor |
|  | Var6 | Monthly Income | 1 = <1000, 2 = 1001-2999, 3 = 3000-5999, 4 = ≥6000 |
|  | Var7 | Education Level * | (ref.= Primary school and below)  Primary school and below: Primary school and below = 0, Junior school = 0, High school = 0, College and above = 0  Junior school: Primary school and below = 0, Junior school = 1, High school = 0, College and above = 0  High school: Primary school and below = 0, Junior school = 0, High school = 1, College and above = 0  College and above: Primary school and below = 0, Junior school = 0, High school = 0, College and above = 1 |
|  | Var8 | Registered of Residence * | (ref.=Rural)  Rural: Rural = 0, Urban = 0  Urban: Rural = 0, Urban = 1 |
|  | Var9 | Living Conditions * | (ref.=Living with others)  Living with others: Living with others = 0, Living alone = 0  Living alone: Living with others = 0, Living alone = 1 |
| Lifestyle information | Var10 | Alcohol Consumption | 1 = No, 2 = Yes |
|  | Var11 | Current Smoker | 1 = No, 2 = Yes |
|  | Var12 | Eating Habits * | (ref.= Vegetarian based)  Vegetarian based: Vegetarian based = 0, Meat-based = 0, Meat-vegetable balance = 0  Meat-based: Vegetarian based = 0, Meat-based = 1, Meat-vegetable balance = 0  Meat-vegetable balance: Vegetarian based = 0, Meat-based = 0, Meat-vegetable balance = 1 |
|  | Var13 | Physical Exercise * | (ref.=Never)  Never: Never = 0, Regular = 0, Irregular = 0  Regular: Never = 0, Regular = 1, Irregular = 0  Irregular: Never = 0, Regular = 0, Irregular = 1 |
| Medical characteristics | Var14 | Presence of Multimorbidity * | (ref.=No)  No: No = 0, Yes = 0  Yes: No = 0, Yes = 1 |
|  | Var15 | Family History of AD | 1 = No, 2 = Yes |
| Measurements tools | Var16 | MoCA | Continuous variable |
|  | Var17 | ADL | Continuous variable |
|  | Var18 | AS-CDM | Continuous variable |

***** The dummy variable was set in hierarchical regression analyses; **MoCA**: Montreal Cognitive Assessment Test; **ADL**: Activities of Daily Living Scale; **AS-CDM**: adherence scale of cognitive dysfunction management
