# Supplementary material for: Correlates of Adherence of Multimodal Non-pharmacological Interventions in Older Adults With Mild Cognitive Impairment: A Cross-Sectional Study
Source: Front Psychiatry. 2022 Jun 3;13:833767. doi: 10.3389/fpsyt.2022.833767 (PMC9210931; doi:10.3389/fpsyt.2022.833767)
Supplement: Supplementary file 3 [file Table_3.DOCX]

Supplementary Material

# Supplementary Material 3

1. **Validity Analysis -- Exploratory Factor Analysis (EFA)**

| **Table S1**. KMO and Bartlett's test (second exploratory factor analysis) | | |
| --- | --- | --- |
| KMO and Bartlett's test | | |
| Kaiser-Meyer-Olkin Measure of Sampling | Adequacy 0.873 | .736 |
| Bartlett's Test of Sphericity | Approx. Chi-square | 5504.788 |
|  | df | 496 |
|  | Sig. | .000 |

| **Table S2**. Questionnaire factor structure for AS-CDM (second exploratory factor analysis) | | | | | | |
| --- | --- | --- | --- | --- | --- | --- |
| Items | Factor1 | Factor2 | Factor3 | Factor 4 | Factor 5 | Factor 6 |
| F7 | 0.940 |  |  |  |  |  |
| F2 | 0.906 |  |  |  |  |  |
| F10 | 0.603 |  |  |  |  |  |
| F1 | 0.598 |  |  |  |  |  |
| F9 | 0.594 |  |  |  |  |  |
| F4 | 0.586 |  |  |  |  |  |
| F8 | 0.543 |  |  |  |  |  |
| F3 | 0.542 |  |  |  |  |  |
| D4 |  | 0.777 |  |  |  |  |
| D2 |  | 0.773 |  |  |  |  |
| D5 |  | 0.768 |  |  |  |  |
| D1 |  | 0.716 |  |  |  |  |
| D6 |  | 0.712 |  |  |  |  |
| D3 |  | 0.596 |  |  |  |  |
| D7 |  | 0.558 |  |  |  |  |
| A5 |  |  | 0.793 |  |  |  |
| A1 |  |  | 0.757 |  |  |  |
| A6 |  |  | 0.738 |  |  |  |
| A4 |  |  | 0.599 |  |  |  |
| A2 |  |  | 0.594 |  |  |  |
| A7 |  |  | 0.463 |  |  |  |
| A3 |  |  | 0.427 |  |  |  |
| B3 |  |  |  | 0.929 |  |  |
| B1 |  |  |  | 0.853 |  |  |
| B4 |  |  |  | 0.507 |  |  |
| B2 |  |  |  | 0.439 |  |  |
| E2 |  |  |  |  | 0.892 |  |
| E3 |  |  |  |  | 0.791 |  |
| E1 |  |  |  |  | 0.612 |  |
| C3 |  |  |  |  |  | 0.838 |
| C1 |  |  |  |  |  | 0.816 |
| C2 |  |  |  |  |  | 0.593 |


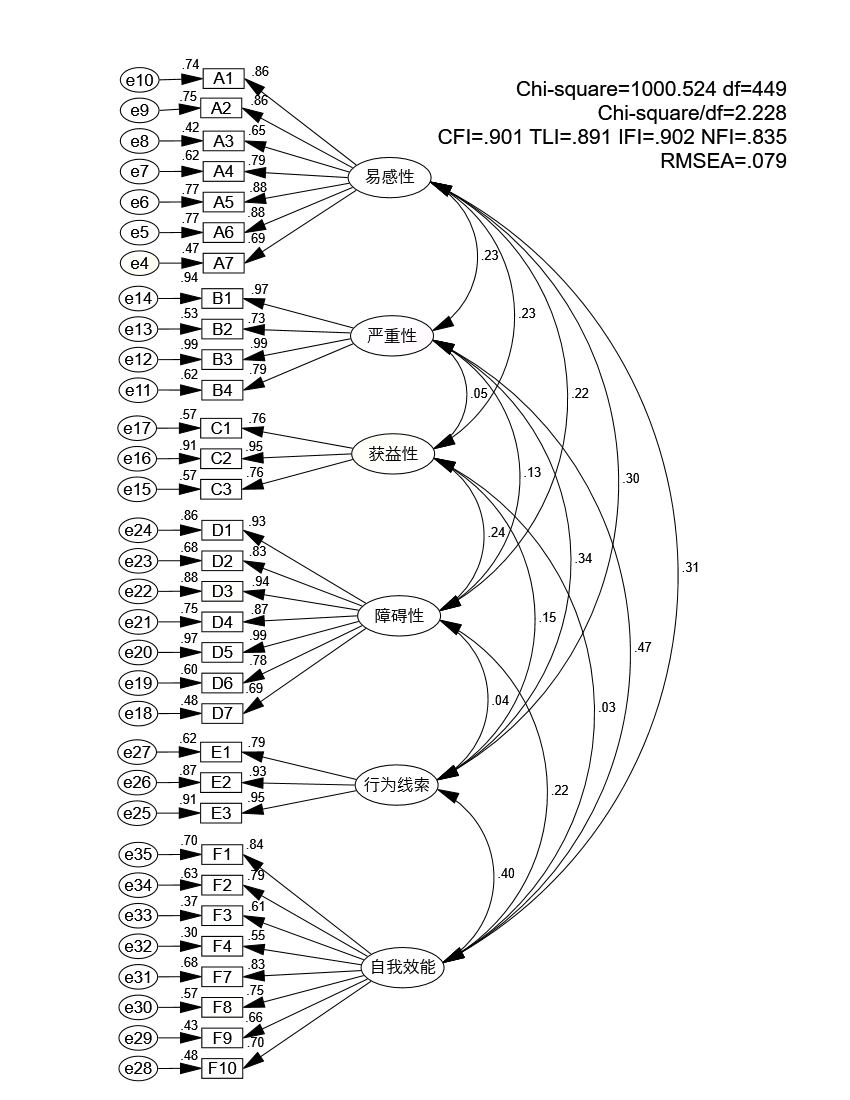


**FigurS3**. Confirmatory factor analysis for AS-CDM (with standardized estimates)

1. **Reliability Analysis**

| **Table S4.** The internal consistency reliability of AS-CDM | | |
| --- | --- | --- |
| Dimension | Cronbach's α | Items |
| Perceived susceptibility | 0.922 | 7 |
| Perceived severity | 0.924 | 4 |
| Perceived benefits | 0.857 | 3 |
| Perceived barriers | 0.953 | 7 |
| Cues to action | 0.918 | 3 |
| Self-efficacy | 0.891 | 8 |
| The overall scale | 0.904 | 32 |
